# A Case of Salivary Fistula

**Published:** 1854-12

**Authors:** F. H. Badger

**Affiliations:** Dentist, of Nashville, Tenn.


### A CASE OF SALIVARY FISTULA.

BY F. H. BADGER, M. D., DENTIST, OF NASHVILLE, TENN.

In July last I was consulted by Mr. D——, aged about twenty-eight years, (a consumptive,) for salivary fistula, produced from a carious upper molar tooth. The saliva proceeded from the left parotid gland, and was discharged externally on that side of the face by two openings, near each other, situated opposite the upper molar teeth, and in the centre of a red scar of uneven surface the size of a half dollar.

The abscess which had formed at the root of the tooth, instead of discharging within the mouth, as is usual in such cases, had pointed upward and outward through the substance of the cheek.

He had consulted two dentists, and one physician, but as he never had pain in the tooth it was not suspected until some weeks before I saw him; it had been extracted at his own request when it was discovered to have been the cause, the discharge of pus having ceased within a few days after the removal. The only annoyance complained of at the time I saw him, was the continued discharge of saliva from the outside of the cheek, which became profuse when eating, insomuch that it was difficult with napkins bound round the jaws to prevent it from running over his clothes at meal times.

Having placed him in my chair, it was by the most careful examination that I was enabled to discover the mouth of the duct, through which the saliva had long ceased to flow. It occurred to me that if this could be reopened and the proper pressure applied over the external artificial openings the saliva would be forced into the proper channel and a cure effected. I was the more encouraged in this hope as the openings were tender and in a condition to heal. Accordingly a very small, smooth, pointed stylet, or gold wire, was selected and by a gentle rotary motion passed in o the duct until it reached the path of the new opening, and on withdrawing it a drop of saliva followed.

A piece of india-rubber of the proper size was then cut from a sheet about one line in thickness, and applied over the artificial openings. This substance was chosen for its aptness to adapt itself to the inequalities of the surface; a pledget of cotton was then placed over this, and a handkerchief tied over the jaws to retain it. The saliva was immediately forced into the proper channel, and ceased altogether to flow externally; the bandage was kept in place until the next day when it was removed and the parts found entirely dry, it was replaced however some time after, and it was advised to be worn some days longer. In the meantime the gentleman left, but it is believed a cure was effected.

Nov. 3, 1854.

